# Epitope determination of immunogenic proteins of *Neisseria gonorrhoeae*

**DOI:** 10.1371/journal.pone.0180962

**Published:** 2017-07-19

**Authors:** Daniel O. Connor, Lena Danckert, Sebastian Hoppe, Frank F. Bier, Markus von Nickisch-Rosenegk

**Affiliations:** 1 Department of Molecular and Cellular Bioanalytics, Fraunhofer Institute for Cell Therapy and Immunology, Branch Bioanalytics and Bioprocesses, Potsdam, Germany; 2 Institute of Biochemistry and Biology, University of Potsdam, Potsdam, Germany; 3 Department of Automation, Fraunhofer Institute for Cell Therapy and Immunology, Branch Bioanalytics and Bioprocesses, Potsdam, Germany; New York State Department of Health, UNITED STATES

## Abstract

*Neisseria gonorrhoeae* is the causative organism of gonorrhoea, a sexually transmitted disease that globally accounts for an estimated 80 to 100 million new infections per year. Increasing resistances to all common antibiotics used for *N*. *gonorrhoeae* treatment pose the risk of an untreatable disease. Further knowledge of ways of infection and host immune response are needed to understand the pathogen-host interaction and to discover new treatment alternatives against this disease. Therefore, detailed information about immunogenic proteins and their properties like epitope sites could advance further research in this area. In this work, we investigated immunogenic proteins of *N*. *gonorrhoeae* for linear epitopes by microarrays. Dominant linear epitopes were identified for eleven of the nineteen investigated proteins with three polyclonal rabbit antibodies from different immunisations. Identified linear epitopes were further examined for non-specific binding with antibodies to *Escherichia coli* and the closely related pathogen *Neisseria meningitidis*. On top of that, amino acids crucial for the antibody epitope binding were detected by microarray based alanine scans.

## Introduction

*Neisseria gonorrhoeae* is one of two pathogenic species within the genus *Neisseria* and is the causative organism of the curable sexually transmitted disease (STD) gonorrhoea that is strictly limited to the human host. The pathogen appears as a gram-negative diplococcus and is, thus, often abbreviated as gonococcus. During infection, the bacteria colonize the urogenital epithelial cells. Since gonococcal infections are asymptomatic in up to 80% of women [1] and 10 to 40% in men [2,3], many patients remain untreated. In consequence, pelvic inflammatory disease (PID) occurs in up to 40% of untreated women [4]. Furthermore, the infection can lead to sterility in men and infertility in women [1]. The WHO estimates up to one million sexually transmitted nonviral infections per day [5]. *N*. *gonorrhoeae* accounts for more than 20% of those infections. Furthermore, a report from the Centers for Disease Control and Prevention (CDC) published in 2014 stated that the three major bacterial STDs have shown substantial increase rates since 2013: Gonorrhoea 5.1%, Chlamydia 2,8% and Syphilis 15,1% [6]. Although reports of STDs can be found in a lot of ancient texts [7], STDs have never been extinguished fully, they have rather been merely controlled during the last decades by the use of antibiotics. Since the advent of antibiotics, however, *N*. *gonorrhoeae* has developed resistances against the majority of commonly used antibiotics leading to a potentially untreatable pathogen in the future [8–11]. The current guidelines from the CDC and the European guideline on the diagnosis and treatment of gonorrhoea in adults recommend the combination of oral azithromycin and injectable ceftriaxone as the last remaining first-line treatment [12,13]. Thus, it is paramount to expand the arsenal in available treatment options sooner rather than later, before the aforementioned antibiotics become ineffective. Hence, a deeper insight into the immunogenic and virulence-associated proteins of *N*. *gonorrhoeae* might help to unveil novel treatment options and nurture new diagnostic devices. We have previously identified six novel immunogenic proteins of *N*. *gonorrhoeae* via phage display while also verifying the immunogenic character of thirteen additional proteins [14]. These proteins were found to be involved in important processes of the pathogens metabolism rendering them highly attractive to our purpose. Therefore, further investigation of the identified proteins is of high relevance.

Consequently, for the present study, those previously identified proteins were investigated for linear epitopes and identified epitopes were subsequently characterised by specificity assays and alanine scans. Epitopes might be utilised for the generation of specific antibodies or for vaccination [15]. By identifying linear epitopes potential vaccine candidates have already been obtained for *Staphylococcus aureus* [16], *Klebsiella pneunomiae* [17] or the dengue virus [18] amongst others. Since the proteins were presented as polypeptide fragments on the phage surface and not as full-length proteins, only the former were analysed, thus significantly reducing the number of overlapping peptides needed for epitope mapping. Additionally, as those polypeptides were originally isolated due to their immunogenic character, it can be safely assumed that the fragments contain the epitope sites.

## Results

### Epitope mapping

**Fig 1:**
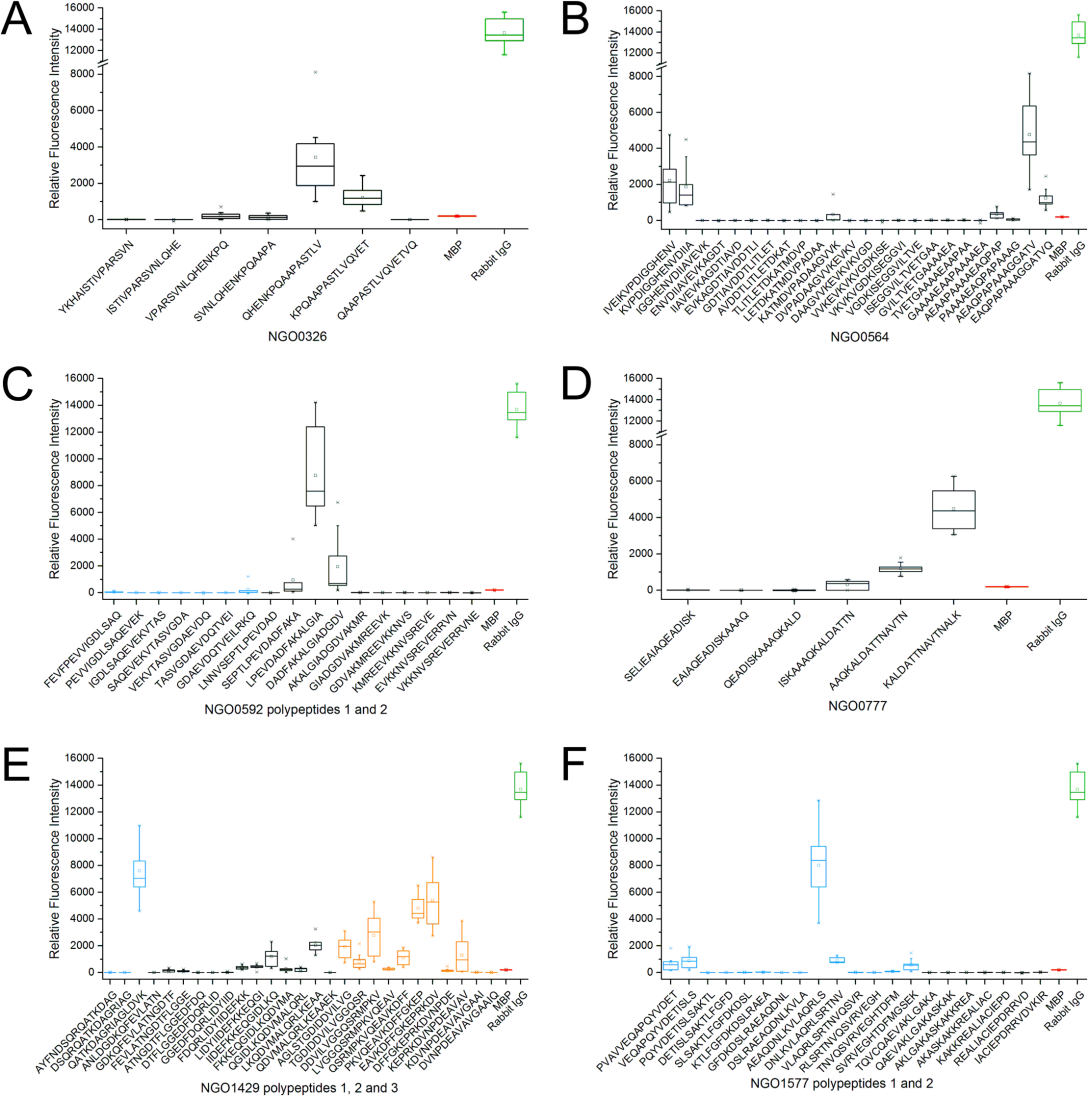
**Epitope mapping (n = 9) of the polypeptides of A) NGO0326, B) NGO0564, C) NGO0592, D) NGO0777, E) NGO1429, F) NGO1577.** The boxes embody 50% of the values, while the whiskers comprise 98% of the data. Outliers are marked with a small x. Median values are indicated as a horizontal line, while the mean values are marked by a small square. Myelin basal protein (MBP) and rabbit Immunoglobulin G (IgG) were included as negative (red) and positive (green) controls. If two or more polypeptides of one protein were examined, boxes are coloured blue for peptides of polypeptide 1, black for the second polypeptide and orange for the third polypeptide.

**Fig 2:**
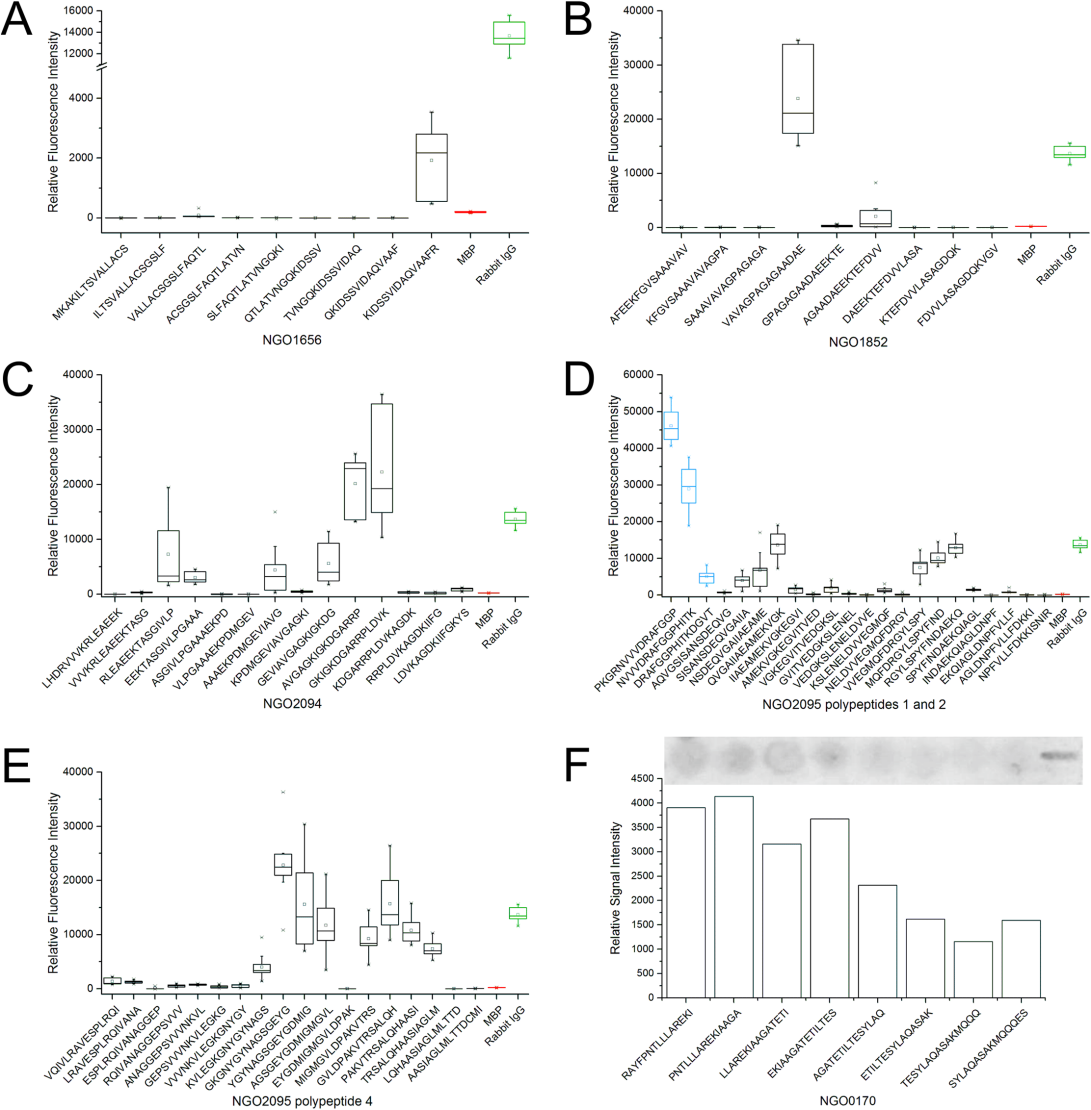
Epitope mapping (n = 9) of the remaining polypeptides. A) NGO1656, B) NGO1852, C) NGO2094, D) NGO2095 polypeptides 1 and 2, E) NGO2095 polypeptide 4, F) NGO0170 via cellulose membrane (Lot# 3542_1). A)–E) The boxes embody 50% of the values, while the whiskers comprise 98% of the data. Outliers are marked with a small x. Median values are indicated as a horizontal line, while the mean values are marked by a small square. Myelin basal protein (MBP) and rabbit Immunoglobulin G (IgG) were included as negative (red) and positive (green) controls. If two polypeptides of one protein were examined boxes are coloured blue for peptides of polypeptide 1 and black for the second polypeptide. F) Colour signals were inverted and analysed by GenePix Pro 7 and visualised as histogram.

Epitope mappings were conducted on microarray slides for all proteins except NGO0170 which was conducted on a cellulose membrane. Based on previously performed phage display analysis polypeptide segments of corresponding proteins were selected and the results are summarized as box-and-whisker plots. These were calculated from merged data obtained by incubation with three different polyclonal rabbit antibodies to *N*. *gonorrhoeae* and three replicates of the immobilised peptide per incubated subarray (n = 9). Results of the specificity controls are given in the supplementary figures (S1 Fig, S2 Fig). Table 1 lists all of the previously identified proteins, states the regions used for epitope mapping, and summarizes the identified epitope sequences [14]. Additionally, the results of the epitope mappings are split evenly between Fig 1 and Fig 2. The former covers epitopes of NGO0326, NGO0564, NGO0592, NGO0777, NGO1429, and NGO1577, while the latter summarizes epitope mappings of NGO1656, NGO1852, NGO2094, NGO2095 polypeptides 1 and 2, NGO2095 polypeptide 4 as well as NGO0170, which was the only epitope mapping to be conducted on a cellulose membrane. The epitope mappings of the remaining proteins featured in Table 1, namely NGO0584, NGO0642, NGO0916, NGO0983, NGO1043, NGO1634, NGO1796, and NGO2139 resulted either in very low signal intensities in the range of the negative control or high variance. Thus, the results are not shown here as no reliable linear epitope sites could be identified in these cases. Specificity controls were generally in the range of the negative controls for the polyclonal antibodies to *E*. *coli* and *N*. *meningitidis*. However, a few exceptions to this were observed. For peptide 0592_2_3 incubation with *N*. *meningitidis* antibody led to signal intensities of approximately 25% as compared to the specific interaction. Peptide 1429_3_1 (AGLSTGDIDDVILVG) harboured approximately 10% of the signal intensity of the original signal for the *E*. *coli* antibody (S1E Fig). The specificity assays for all peptides of NGO2095 displayed no non-specific interaction except for 2_12 and 2_13 with 16% and 10% of the original intensity after incubation with the anti-*N*. *meningitidis* antibody.

**Table 1:** Immunogenic proteins identified by phage display in a previous work [14].

*N*. *gonorrhoeae* locus tag	Protein name	Predicted localisation PSORTb	Region used for epitope mapping and total length of amino acid sequence	Identified epitope peptide number and sequence
NGO0170	ABC transporter, ATP-binding protein	Cytoplasmic membrane	205−245 (251)	Peptide 1_3 LLAREKIAAGATETI
NGO0326	RNA-binding protein Hfq	Cytoplasm	57−93 (97)	Peptide 1_5 QHENKPQAAPASTLV
NGO0564	dihydrolipoamide acetyltransferase	Cytoplasm	3−106 (539)	Peptide 1_1 IVEIKVPDIGGHENV Peptide 1_23 AEAQPAPAAAGGATV
NGO0584	50S ribosomal protein L9	Cytoplasm	38−81 (150)	-
NGO0592	trigger factor	Cytoplasm	111−149; 239−286 (437)	Peptide 2_3 LPEVDADFAKALGIA
NGO0642	tRNA pseudouridine synthase B	Cytoplasm	1−88 (306)	-
NGO0777	DNA-binding protein Hu	Cytoplasm	4−37 (89)	Peptide 1_6 KALDATTNAVTNALK
NGO0916	dihydrolipoamide succinyltransferase	Cytoplasm	1−91 (393)	-
NGO0983	outer membrane protein H.8	Outer membrane	23−80 (88)	-
NGO1043	hypothetical protein	Periplasm	35−88 (114)	-
NGO1429	molecular chaperone DnaK	Cytoplasm	144−166; 209−269; 328–379; 517–592 (642)	Peptide 1_3 QATKDAGRIAGLDVK Peptides 2_9 IIDEFKKEQGIDLKQ Peptide 3_7 EAVKDFFGKEPRKDV
NGO1577	outer membrane protein PIII	Outer membrane	76–142; 182–220 (236)	Peptide 1_2 VEQAPQYVDETISLS Peptide 1_9 AEAQDNLKVLAQRLS
NGO1634	hypothetical protein, putative phage associated protein	Cytoplasm	116–148 (183)	-
NGO1656	conserved hypothetical protein	Unpredictable	1–44 (288)	Peptide 1_9 KIDSSVIDAQVAAFR
NGO1796	ribosome recycling factor	Cytoplasm	32–130 (185)	-
NGO1852	50S ribosomal protein L7/L12	Unpredictable	26–71 (123)	Peptide 1_4 VAVAGPAGAGAADAE
NGO2094	chaperonin 10 kDa subunit	Cytoplasm	6–71 (96)	Consensus of peptides 3 and 4 EEKTASGIVLP Consensus of peptides 10 and 11 GKIGKDGARRP
NGO2095	chaperonin 60 kDa subunit	Cytoplasm	33–55; 145–231; 301–401; 440–521 (544)	Peptide 1_1 PKGRNVVVDRAFG Consensus of peptides 2_3 and 2_4 DEQVGAIIAEA Peptide 2_13 FDRGYLSPYFI Peptide 4_10 YGYNAGSGEYGDMIG Peptide 4_14 GVLDPAKVTRSALQH
NGO2139	genome-derived Neisserial antigen 1946	Cytoplasmic membrane	123–231 (288)	-

Only those parts of each protein presented on the phage surface were examined for epitopes by epitope mapping.

### Alanine scan

All identified epitopes summarized in Table 2 were further characterized by alanine scan to determine amino acids that are critical for antibody-epitope binding. Considerable drops of signal intensity upon substitution with alanine indicate crucial amino acids for binding of the antibody to its epitope. The results of the alanine scans are included in Table 2 with additional information about the conservation of the epitope sequence. The corresponding Box-Whisker-Plots can be found in the supplementary figures (S3 Fig, S4 Fig and S5 Fig).

**Table 2:** Summary of the alanine scans to determine crucial amino acids for antibody-epitope binding.

Name	Original sequence	Loss of binding upon substitution of residues	Conserved in (100% identity with BLAST)
Conserved in Neisseria or on species level and signal decreases > 90%			
NGO0777_1_6	KALDATTNAVTNALK	D (Pos. 4) 96%, T (Pos. 6) 99%	*Neisseria*
NGO1577_1_9	AEAQDNLKVLAQRLS	E (Pos. 2) 99 %; D (Pos. 5) 61%; N (Pos. 6) 99%; L (Pos. 10) 63%; R (Pos. 13) 99%	*N*.* gonorrhoeae*, *N*.* meningitidis*
NGO1852_1_4	VAVAGPAGAGAADAE	P (Pos. 6) 82%; G (Pos. 8) 78%; G (Pos. 10) 90%; D (Pos. 13) 72%	*N*.* gonorrhoeae*, *N*.* meningitidis*
NGO2094_1_3c	EEKTASGIVLP	T (Pos. 4) 88%; S (Pos 6.) 76%; G (Pos. 7) 97%	*Neisseria*
NGO2094_1_10c	GKIGKDGARRP	I (Pos. 3) 90%; R (Pos. 9) 97%	*Neisseria*
NGO2095_4_10	YGYNAGSGEYGDMIG	E (Pos. 9) 99%	*N*.* gonorrhoeae*
Conserved in the kingdom bacteria or family Neisseriaceae and signal decreases > 90%			
NGO0564_1_1	IVEIKVPDIGGHENV	DIGGH (Pos. 8−12) 99%	Neisseriaceae
NGO1429_1_3	DAGRIAGLDVK	GLD (Pos. 7−9) and K (Pos. 11) 99 %; V (Pos. 10) 83%	Bacteria
NGO1429_3_7	EAVKDFFGKEPRKDV	D (Pos. 5) 90%; F (Pos. 6) 95%; F (Pos. 7) 99%; G (Pos. 8) 90%	Proteobacteria
NGO2095_2_13c	FDRGYLSPYFI	D (Pos. 2) 99%; GY (Pos. 4 and 5) 99%; L (Pos. 6) 84%; SP (Pos. 7 and 8) 99%	Bacteria
Conserved in N. gonorrhoeae or N. meningitidis and signal decreases < 90%			
NGO0326_1_5	QHENKPQAAPASTLV	A (Pos.8) 67%, A (Pos. 9) 63%, L (Pos. 14) 83%	*Neisseria gonorrhoeae*
NGO0564_1_23	AEAQPAPAAAGGATV	P (Pos. 7) 52%; G (Pos. 11) 63%; T (Pos. 14) 79%	*Neisseria gonorrhoeae*
NGO1577_1_2	VEQAPQYVDETISLS	Q (Pos. 3) 68%; A (Pos. 4) 63%	*N*.* gonorrhoeae*, *N*.* meningitidis*
Conserved in the order Neisseriales or the family Neisseriaceae and signal decreases < 90%			
NGO0592_2_3	LPEVDADFAKALGIA	D (Pos. 5) 70%	Neisseriales
NGO2095_1_1	PKGRNVVVDRAFG	D (Pos. 9) 53%; R (Pos. 10) 71%	Neisseriaceae
NGO2095_2_3c	DEQVGAIIAEA	G (Pos. 5) 75%; I (Pos. 7) 88%	Neisseriaceae
Ambiguous results or no quantification applicable			
NGO0170_1_3	LLAREKIAAGATETI	No quantitative evaluation possible	Neisseria
NGO1656_1_9	SVIDAQVAAFR	low overall signal intensities	*Neisseria*
NGO2095_4_14	GVLDPAKVTRSALQH	No considerable loss of binding	Neisseriales

Epitopes are named by their protein origin, the polypeptide region and the peptide which was determined as epitope site. Consensus sequences are labelled with c. Crucial amino acids are underlined in the original sequence and listed in the third column with their percentage loss in comparison to the mean value of the remaining peptides. Furthermore, conservation of the epitope sequence shows the specificity of the epitope region. BLAST results with 100% query cover and 100% identity were defined as conservation parameter.

The alanine scan results can be divided into five groups. Various amino acid substitutions in the sequences of the identified epitopes resulted in signal decreases of more than 90% compared to the mean signal intensities of the remaining peptides. The epitopes of the first group are conserved in the genus *Neisseria* or on species level and showed signal decreases > 90% for substitutions of certain amino acids. This was true for NGO1577_1_9, NGO1852_1_4, NGO2095_4_10, NGO0777_1_6, NGO2094_1_3c and NGO2094_1_10c. The first two are both conserved in *N*. *gonorrhoeae* or *N*. *meningitidis* and contained five or four crucial residues. NGO2095_4_10 is solely conserved in *N*. *gonorrhoeae* and contains one crucial residue. The remaining three are all conserved in the genus Neisseria and substitution of individual amino acids led to an almost complete signal loss.

The second group merges the epitopes that showed signal decreases of > 90% and are conserved in the kingdom bacteria or the family Neisseriaceae and thus are not specific for *Neisseria*. These epitopes were NGO0564_1_1, NGO1429_1_3, NGO1429_3_7 and NGO2095_2_13c. All of these epitopes revealed a sequence of 4 to 5 consecutive amino acids where substitution of single amino acids led to an almost complete signal loss.

The third group consists of epitopes that are conserved in the species *N*. *gonorrhoeae* and *N*. *meningitidis* but substitutions of single amino acids resulted in signal decreases of < 90% compared to the mean signal intensities of the remaining peptides. This applied to NGO0326_1_5, NGO0564_1_23, and NGO1577_1_2. Substitution of amino acids led to a distinct signal decrease of 40–83%, although the signal drop was not as fundamental as for epitopes in group one and two.

The fourth group includes epitopes that are conserved in the order Neisseriales or the family Neisseriaceae and showed signal decreases < 90% during alanine scan. NGO0592_1_3, NGO2095_1_1 and NGO2095_2_3c fall into this category. The remaining epitopes, NGO1656_1_9 and NGO2095_4_14 showed ambiguous results and comprised group 5. Residues essential for binding could not be determined.

The alanine scan for NGO0170 was performed with immobilised peptides on a cellulose membrane (S6 Fig). Quantification was not equally applicable as colour spots show different intensities. Peptides 1 to 3 and peptides 7 to 10 showed a reduced colour reaction upon substitution. Those amino acids could be relevant for antibody binding. In addition, the epitope is part of a membrane-bound protein and conserved in the genus *Neisseria*.

### Mapping to protein models

All identified epitopes and crucial amino acids for antibody binding were mapped to predicted protein models of each protein. Furthermore, the phage presented polypeptides that were identified in a previous work and investigated for epitope mapping are marked in the models. All models were checked by QMEAN for their reliability (Table 3). All QMEAN values lay above the cutoff of 0.4 except for the model of NGO1577. Additionally, Table 3 includes information about the closest structural homologues of each examined protein.

**Table 3:** Summary of obtained bioinformatic properties of the protein models.

Protein locus tag	QMEAN value	Closest structural homologue	PDB ID	Host organism	Quarternary structure	Epitope included in homologous structure	Reference
NGO0170	0.652	ABC transporter	2NQ2	*Haemophilus influenza*	heterotetramer	yes	[19]
NGO0326	0.630	Hfq	4NL2	*Lysteria monocytogenes*	homohexamer	no	[20]
NGO0564	0.487	catalytic domain of dihydrolipoamide acetyltransferase	4N72	*Escherichia coli*	homotrimer	no	[21]
NGO0592	0.618	trigger factor protein	1W26	*Escherichia coli*	monomer	yes	[22]
NGO0777	0.586	DNA binding protein	1P71/ 1P51	*Anabeana* sp./ *Staphylococcus aureus*	homodimer	yes	[23,24]
NGO1429	0.675	chaperone HSP70	4JNE	*Escherichia coli*	homodimer	yes	[25]
NGO1577	0.308	metallopeptidase	3IUU	*Mesorhizbium* sp. BNC1	monomer	no	[26]
NGO1656	0.414	SurA-like chaperone	3RFW	*Campylobacter jejuni*	homodimer	yes	Not published
NGO1852	0.560	ribosomal protein L12	1DD3	*Thermogota maritima*	heterotrimer	yes	[27]
NGO2094	0.434	mitochondrial chaperone/ GroEL-GroES complex	4PJ1/ 1PCQ	*Homo sapiens/* *Escherichia coli*	hetero-28-mer hetero-21-mer	yes	[28,29]
NGO2095	0.734	GroeEL protein	1KP8	*Escherichia coli*	homo-14-mer	yes	[30]

**Fig 3:**
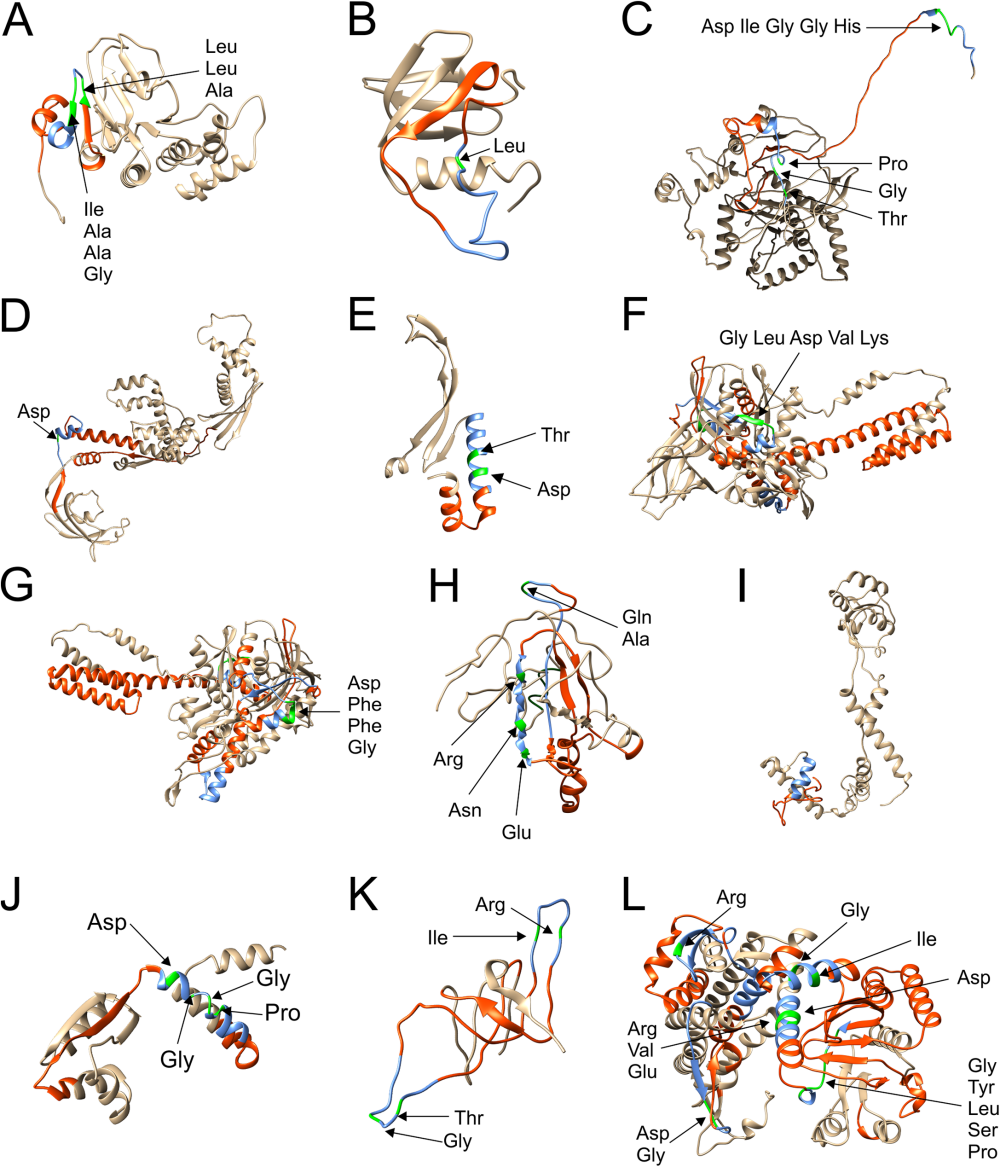
3-dimensional monomeric structure models of the immunogenic proteins. Isolated polypeptides determined in a previous work are marked in orangered, identified epitopes are highlighted in blue, amino acids crucial for antibody binding are coloured in green and individual amino acids are labeled by an arrow. A) NGO170 B) NGO0326 C) NGO0564 D) NGO0592 E) NGO0777 F) NDO1429 G) NGO1429 with a 180° turn around the y-axis and 45° turn around the x-axis. H) NGO1577 I) NGO1656 J) NGO1852 K) NGO2094 L) NGO2095.

**Fig 4:**
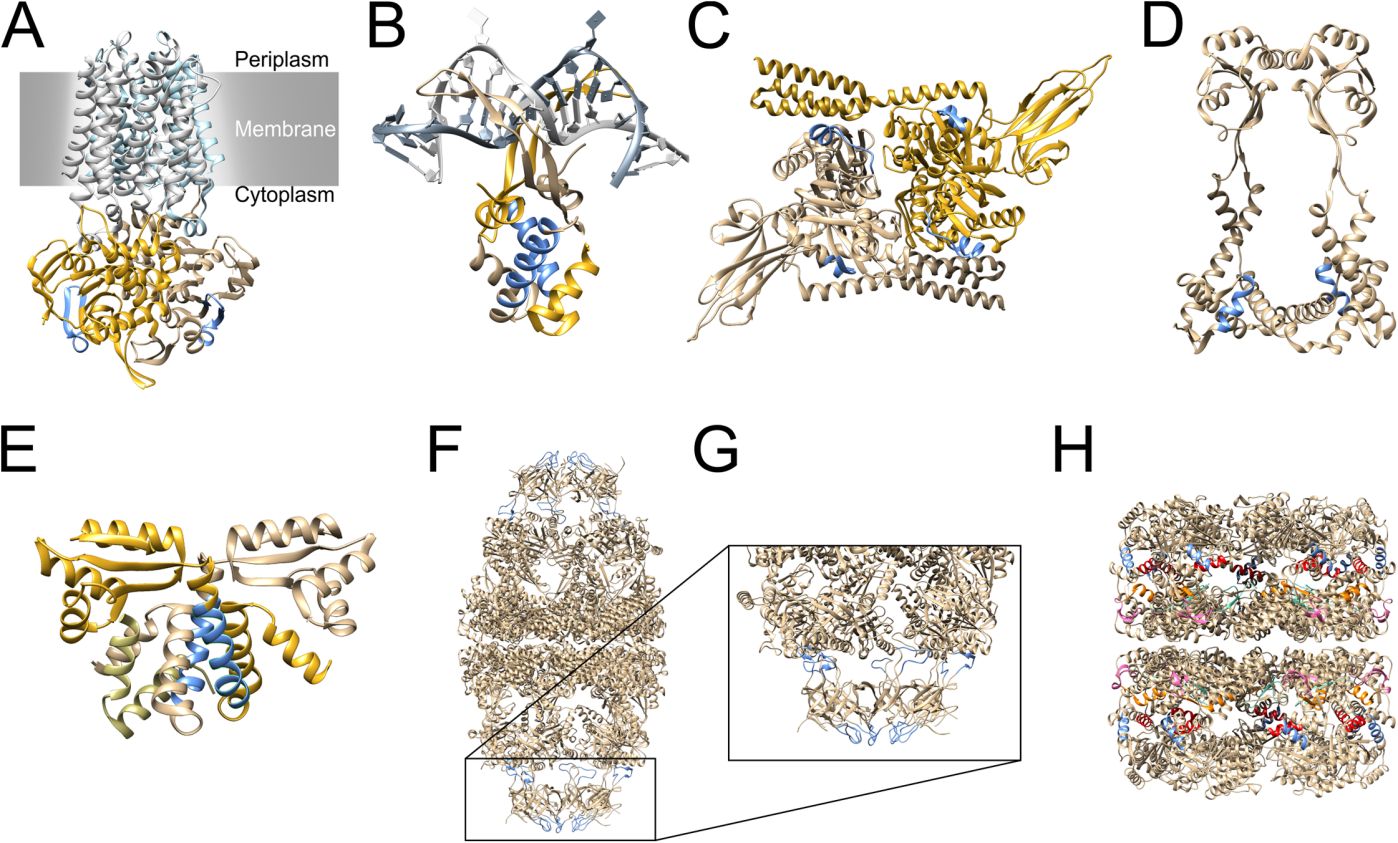
3-dimensional structure models of the closest structural homologues. A) Heterotetramer of a structural homologue of NGO0170 embedded in the cytoplasmic membrane (PDB ID: 2NQ2). B) Homodimer of structural homologue of NGO0777 bound to a DNA double strand (PDB ID: 1P51). C) Homodimer of a structural homologue of NGO1429 (PDB ID: 4JNE). D) Homodimer of a structural homologue of NGO1656 (PDB ID: 3RFW). E) Heterotrimer of a structural homologue of NGO1852 (PDB ID: 1DD3). F) Hetero-28-mer of a structural homologue of NGO2094 (PDB ID: 1PCQ). G) Zoomed in part of the Hetero-28-mer of F. H) Homo-21-mer of a structural homologue of NGO2095 (PDB ID: 1KP8). Identified epitopes are highlighted in blue. Identified epitopes of NGO2095 are marked in red, blue, orange, green and pink.

NGO0170 was predicted to be localised in the cytoplasmic membrane. The identified polypeptide was located at the C-terminal end of the protein spanning three α-helices and two β-sheets (Fig 3A). The epitope region was localised in the middle of the polypeptide and is highlighted in blue. The two determined crucial regions for antibody binding were located just before and after a loop. The closest structural homologue consists of two identical chains that are embedded in the membrane and two homologous chains of NGO0170 extending into the cytoplasm (Fig 4A). Likewise, the polypeptide of NGO0326 was located at the C-terminal end of the protein and encompassed a β-sheet and random coil structures (Fig 3B). The identified epitope was localised at the C-terminal end of the polypeptide. Alignments of all sequences of the structural homologues did not include the epitope’s sequence. Hence, only the monomeric model is shown. The identified polypeptide of NGO0564 had a length of 102 amino acids and was located at the N-terminal end of the protein (Fig 3C). Alignments of available structural homologues in the PDB database included only amino acids starting from residue number 287. Therefore, most of the polypeptide structure was modelled as random coil. The identified epitopes were located at the N-terminal end and one at the C-terminal end of the polypeptide. Both epitopes were located in that part of the structure model that was not covered by alignments. Mapping of the epitope to the multimeric model was not applicable since the corresponding part was not covered by the homologous models. The two identified polypeptides of NGO0592 were located in the middle of the amino acid sequence (Fig 3D). The identified epitope included an α-helix and random coil structures. The identified polypeptide of NGO0777 was located at the N-terminal end of the protein (Fig 3E). Structural motifs of the polypeptide were largely α-helices. The epitope region and two crucial amino acids for antibody binding were located at the C-terminal end of the polypeptide. Epitopes were also identified in homodimeric model (Fig 4B). Four identified polypeptides for NGO1429 were located in different parts of the protein (Fig 3F and 3G). The two epitopes that were successfully analysed by alanine scans are highlighted in the multimeric model (Fig 4C). Most of NGO1577 secondary structure was modelled as random coil structure (Fig 3H). The obtained QMEAN value was 0.308 resulting in a free modelled protein target. The only epitope contained in the IEDB database was located separated from the examined polypeptides [31,32]. This epitope is coloured in dark green in the middle of the snap-shot of the protein model. The polypeptide isolated for NGO1656 was located at the N-terminal end of the protein and included an α-helix and random coil structures (Fig 3I). The identified epitope was determined at the C-terminal end of the polypeptide. The epitope was located at the bottom part when considering the snap-shot of the multimeric model (Fig 4D). The isolated polypeptide of NGO1852 stretched across three α-helices and one β-sheet in the middle of the protein’s primary structure (Fig 3J). The identified epitope is located in the middle of the phage presented polypeptide. The quarternary structure of the homologue is assembled with two identical subunits of the homologous protein of NGO1852 and another subunit (Fig 4E). Many different parts of both proteins NGO2094 and NGO2095 were identified as immunogenic polypeptides by phage display in a previous work. Hence, large parts of the proteins were examined for epitopes. The two identified epitopes of NGO2094 were located at the accessible outer regions of the protein (Fig 3K). Crucial amino acids for antibody binding were determined just before and behind the loops of both epitopes. The two obtained multimeric structures of a chaperon complex differ mainly in number and localisation of the GroES chains. While GroES chains are assembled on both sides of the human protein, they are only associated on one side of the *E*. *coli* protein. Six epitope regions were identified for NGO2095 (Fig 3L). Most epitopes were accessible in the protein model and not buried inside. The identified six epitopes are coloured differently in the multimeric model (Fig 4H). Two epitopes (blue and pink) are presented on the surface of the protein model while the other epitopes seemed to be buried in the displayed X-ray diffraction snap-shot.

## Discussion

Nineteen immunogenic proteins of *N*. *gonorrhoeae* identified in a previous work [14] were examined for linear epitopes by microarray epitope mapping in this study. Identification of linear epitopes by peptide microarray is a well-established technique which has been applied successfully for the determination of linear epitope regions from other human pathogens [33,34]. Distinct linear epitopes were identified with three polyclonal rabbit antibodies for 11 of the examined 19 proteins. Hence, the identified epitopes appear to possess an immunodominant character that is recognised and processed by the immune response of individual groups of experimental animals, namely rabbits. Notably, linear epitopes were identified for 58% of the examined proteins, contrary to the general occurrence of linear epitopes (10%) compared to structural epitopes (90%) in nature [35]. This high number of identified linear epitopes arose most likely through the preceding selection of phage presented polypeptides, since most presented polypeptides represent only partial regions of the corresponding full-length protein. Therefore, tertiary and quaternary structures are most likely not formed as in the full-length protein so that linear epitopes are bound by immobilised antibodies to a higher extent and amplified through consecutive panning rounds. Novel linear epitopes of specific antigens could play an essential role for the development of diagnostics or suitable vaccines [15,36] e. g. for the generation of monoclonal antibodies which than could be applied in a diagnostic test or as part of a vaccine used for active or passive immunisation. For both applications specific targets are crucial. Hence, we examined potential cross reactivities to *N*. *meningitidis* and *E*. *coli* antibodies which resulted in specific binding for most of the identified epitopes. Only two epitopes, NGO0592_1_3 (LPEVDADFAKALGIA) and NGO2095_2_13c (DEQVGAIIAEA) showed partial non-specific binding after incubation with the anti-*N*. *meningitidis* antibody. Non-specific binding amounted to 25% and 10% compared to the original signals. All identified linear epitope sequences were checked for conservation by BLAST using blastp suite. Homology of NGO0592_1_3 was high throughout many species of Neisseriales including *N*. *meningitidis* with 100% identity. NGO2095_2_13c was conserved among many bacteria (see Table 2). On the contrary, NGO0326_1_5, NGO0564_1_23 and NGO2095_4_10 showed alterations in their sequence even for closely related organisms like *N*. *meningitidis* and other *Neisseria* species, indicating highly specific epitope regions for *N*. *gonorrhoeae*. These bioinformatic findings concur with the experimental results that entailed no binding to antibodies reactive to other bacteria. The other epitopes were conserved either in *N*. *gonorrhoeae* and *N*. *meningitidis*, *Neisseria*, Neisseriaceae or Neisseriales. Three identified epitopes (NGO1429_1_3, NGO1429_3_7 and NGO2095_2_13c) were conserved in the phylum of Proteobacteria or in all bacteria.

Epitope mappings for NGO0584, NGO0916, NGO1043, NGO1634 and NGO1796 only showed distinct linear epitopes for the antibody used for the initial screenings of the DNA phage libraries (data not shown). However, incubation with three antibodies yielded widely varying signal intensities although all three polyclonal antibodies were capable to detect the corresponding full-length proteins in a previous study [14]. Presumably, the full-length proteins are immunodominant, however, the polyclonal immune response was triggered by different epitopes. Therefore, it is conceivable that different epitope regions were predominantly addressed by the polyclonal antibody solutions that were obtained through various immunisations. Consequently, individual epitope regions during epitope mapping are not recognised by each of the polyclonal antibody solutions, while the intact full-length proteins are. Hence, although the five full-length proteins showed immunodominant character, the herein identified epitopes did not appear to be immunodominant. In contrast, epitope mapping of the examined part of NGO0983 showed increased signal intensities for seven of the twelve overlapping peptides compared to the negative control (data not shown). NGO0983 is annotated as outer membrane protein H.8 that is built up by a tandem repeat of the sequence AAEA* in which “*” is either Alanine or Proline. Binding regions of the polyclonal antibodies were thereby located in the tandem repeat area. All identified epitopes found in this study have not been described in literature before. However, different epitope regions of some examined proteins had been deposited at the IEDB database: a homologous protein of NGO0564 (*N*. *meningitidis* T-cell epitope DDTLITLETDKATM [37]), NGO0916 (T-cell epitope DEILIDIETDKVVL [37]), a homologue of the repetitive protein NGO0983 (*N*. *meningitidis* B-cell epitope EATPAAEAPASEAPAAEAAP [38]) and NGO1577 (B-cell epitope WKNAYFDK [32]). The bigger part of the examined proteins was predicted to be located in the cytoplasm and hence, epitope accessibility for detection and antibody binding would be hampered. Yet, several of these cytoplasmic proteins were found to be surface exposed (e. g. homologue of NGO1852 [39], compare Connor *et al*. [14]) which results in intriguing targets for further investigations.

Subsequent examination of the identified epitope regions ought to provide an indication of the suitability of these peptides for therapeutic or diagnostic purposes. Therefore, recombinant monoclonal antibodies need to be generated against the epitope peptides and the potential to detect intact or lysed bacterial cells ought to be investigated. Antibodies capable of detecting or flagging the bacterial cell could well be applied to the development of a potential diagnostic tool or serve as a novel therapeutic approach. As compared to recombinant protein production, short epitope peptides benefit from their easy and fast fabrication in large quantities via peptide synthesis. Thus, the identified epitopes could be utilised for serological screenings, if they prove to be immunodominant in humans. Furthermore, epitope peptides have been successfully used for the development of vaccines before [15]. Nevertheless, the results presented herein, were generated using rabbit sera only, leaving a number of aspects to be investigated for further conclusions.

## Material and methods

### Antibodies

Three different polyclonal rabbit antibodies against *N*. *gonorrhoeae* (Acris BP1050 Lot# 2B04511 and Lot# 7A03112; Abcam ab19962 Lot# GR204640-1) were deployed as primary antibodies individually for microarray experiments. One of those polyclonal antibodies (Acris BP1050 Lot# 2B04511) was used for the approaches with cellulose membranes. Polyclonal rabbit antibodies to *Escherichia coli* (Abcam 137967 Lot# GR129883-2) and *Neisseria meningitidis* (AbD Serotec 6600–5906 Lot# 281111) were used to examine specificity of the epitope sites. All polyclonal rabbit antibodies mentioned before were generated by immunisation of whole inactivated cells and subsequent purification by protein A chromatography and were commercially available as polyclonal IgG antibodies. A polyclonal goat antibody to rabbit IgG conjugated with Chromeo546 (Abcam ab60317 Lot# GR35519-8) was applied as a secondary antibody in the microarray experiments enabling a fluorimetric readout. A polyclonal goat antibody to rabbit IgG conjugated with HRP (Abcam, ab6721, Lot# GR3175-3) was used as secondary antibody for the cellulose membrane approach.

### Epitope mapping by microarray technique

Immunogenic proteins that have been identified by phage display in a previous work [14] were used for epitope mapping (Table 1). The polypeptide fragments presented during phage display were divided into overlapping 15-mer oligopeptides in silico with an overlap of eleven and an offset of four amino acid residues. If more than one polypeptide fragment per protein had been identified during phage display, each part was used independently for oligopeptide generation. Peptide synthesis and coupling to microarray slides was conducted by JPT Peptide Technologies GmbH. Microarray slides featured three identical subarrays, with each peptide present as a triplicate per subarray. Incubation was performed by applying a Proplate 3-well chamber system to enable individual incubation of each subarray with different antibodies in parallel, resulting in n = 9 replicates for each examined peptide.

The slides were blocked initially with 2% dried milk powder in phosphate buffered saline (PBS) with 0.05% Tween-20 (MPBST) for 3 h, washed five times with PBS with 0.05% Tween-20 (PBST) and incubated with appropriate primary antibodies at 4°C overnight with gentle agitation (40 rpm), followed by a washing step before the secondary antibody was applied for 2 h in the dark at 40 rpm. The slides were washed three times with PBST and rinsed with a constant flow of H_2_O_dest_. Subsequently, the slide was dried by nitrogen flow and scanned. The same incubation procedure was used as a control for non-specific secondary antibody binding except that the primary antibody was replaced by PBS during incubation. An Axon GenePix 4300A laser scanner (Molecular Devices) was used for scanning the incubated slides. The scanner was operated with the following settings: 532 nm laser, PMT gain 400, 40% laser power, lines to average 1, 10 μm resolution and standard green emission filter at 532 nm. In parallel, potential epitope peptides and surrounding peptides with increased signal intensities compared to the other peptides were examined for non-specific binding with polyclonal antibodies to *E*. *coli* and *N*. *meningitidis*.

### Alanine scan

Individual peptides and consensus sequences of consecutive peptides showing distinct signal intensities during epitope mapping were chosen for subsequent alanine scan. The modified peptides were produced and immobilised onto glass slides by JPT Peptide Technologies GmbH. The array design was identical to the epitope mapping, featuring three identical subarrays with each peptide in triplicate per subarray. Incubation procedure, scanning and analysis were performed as described above for epitope mapping.

### Microarray analysis

The median of the fluorescence intensity of each spot was used for microarray analysis. Each spot was corrected by its local background (median F532 − B532). Signals of the control chamber (PBS and secondary antibody) were subtracted from the corresponding spot signals incubated with primary and secondary antibody to obtain relative fluorescence intensities (RFI) and to exclude non-specific binding of the secondary antibody.

### Epitope mapping and alanine scan via cellulose membrane

The epitope mapping and alanine scan of NGO0170 were performed with cellulose membranes instead of using microarray technique. The phage presented polypeptide (identified in previous work) was divided into peptides of 15 amino acids in length equivalent to the microarray epitope mapping. Peptide synthesis was conducted by JPT Peptide Technologies GmbH directly on the cellulose membrane. Incubation of the membranes for both epitope mapping and alanine scan were performed as follows. All incubation steps were carried out on a horizontal shaker at 40 rpm. First, the membrane was incubated with methanol for 5 min, followed by 3 x 3 min washing with TBS-T (50 mM Tris, 137 mM NaCl, 2.7 mM KCl, pH 8.0 adjusted with HCl, 0.05% Tween 20). Subsequently, the membrane was blocked with 2% skim milk powder in TBS-T (2% MTBS-T) at 4°C overnight or for 2 h at room temperature. Next, the membrane was incubated with 4 μg mL^−1^ of the primary antibody for 3 h and washed with TBS-T (3 x 5 min). Thereupon, the membrane was incubated with 1 μg mL^−1^ of secondary antibody for 2 h. Antibody binding was detected by incubation with substrate (0.1% 3,3‘-Diaminobenzidin (DAB) in TBS, pH 7.3, 100 mM Imidazol, 0.01% H_2_O_2_) for 10 min. Subsequently, the membrane was washed with water (3 x 10 min) and incubated in H_2_O_dest_ overnight to decrease the background. Colour intensities were analysed by inverting the image and using GenePix Pro 7. The identified peptide region was further characterized by alanine scan. The alanine scan was conducted with the same parameters as the epitope mapping.

### Bioinformatics

Localisation prediction was performed with PSORTb v3.0.2 [40]. Visualisation and editing of sequence data was carried out with Geneious Pro 6.1.8. Identified epitopes were compared to deposited epitopes in IEDB database [31]. 3-dimensional structures were predicted using I-TASSER [41–43] and models were visualised with Chimera [44]. Predicted models were selected by their C-score, which is a confidence score used by I-TASSER to estimate the quality of a predicted model. The C-score usually ranges between −5 and 2. Higher C-score values represent models with a higher confidence. The model reliability was additionally checked by QMEAN [45]. Models with QMEAN values < 0.4 are considered to be free modelled targets, while QMEAN values > 0.4 are regarded to be template based models [46]. Linear epitope sequences were checked for conservation by BLAST [47]. Evaluation of results was performed by Microsoft Excel and OriginPro 9.1 G (Originlab).

## Supporting information

S1 Fig **Specificity controls of the identified peptides with epitope features of A) NGO0326, B) NGO0564, C) NGO0592, D) NGO0777, E) NGO1429, F) NGO1577.** Almost all peptides showed specific binding for anti-*N*. *gonorrhoeae* antibodies. Only two peptides of NGO0592 (C) showed unspecific binding to the polyclonal anti-*N*. *meningitidis* antibody correlating to approximately 25% of the signal obtained through specific binding. Minimal signal intensities could also be seen for two peptides of NGO1429 for the anti-*E*. *coli* antibody correlating to maximal 10% of the signal obtained through specific binding.
(TIF)

S2 Fig Specificity controls of the identified peptides with epitope features of the remaining polypeptides. A) NGO1656, B) NGO1852, C) NGO2094, D) the first three potential epitopes NGO2095, E) further two potential epitopes NGO2095. Almost all peptides showed specific binding for anti-*N*. *gonorrhoeae* antibodies. Only two peptides of NGO2095 showed signal intensities for the anti-*N*. *meningitidis* antibody correlating to approximately 10% of the signal obtained through specific binding.
(TIF)

S3 Fig **Alanine scans of the identified epitopes of A) NGO0326_1_5, B) NGO0564_1_1, C) NGO0564_1_23, D) NGO0592_1_3, E) NGO0777_1_6.** The boxes embody 50% of the values, while the whiskers comprise 98% of the data. Outliers are marked with a small x. Median values are indicated as a horizontal line, while the mean values are marked by a small square. Myelin basal protein (MBP) and rabbit Immunoglobulin G (IgG) were included as negative (red) and positive (green) controls. Boxes of substituted amino acids causing considerable signal drops compared to the remaining peptides are coloured in orange.
(TIF)

S4 Fig **Alanine scans of the identified epitopes of A) NGO1429_1_3, B) NGO1429_3_7, C) NGO1577_1_2, D) NGO1577_1_9, E) NGO1852_1_4.** The boxes embody 50% of the values, while the whiskers comprise 98% of the data. Outliers are marked with a small x. Median values are indicated as a horizontal line, while the mean values are marked by a small square. Myelin basal protein (MBP) and rabbit Immunoglobulin G (IgG) were included as negative (red) and positive (green) controls. Boxes of substituted amino acids causing considerable signal drops compared to the remaining peptides are coloured in orange.
(TIF)

S5 Fig **Alanine scans of the identified epitopes of A) NGO2094_1_3c, B) NGO2094_1_10c, C) NGO2095_1_1, D) NGO2095_2_3c, E) NGO2095_2_13c, F) NGO2095_4_10.** The boxes embody 50% of the values, while the whiskers comprise 98% of the data. Outliers are marked with a small x. Median values are indicated as a horizontal line, while the mean values are marked by a small square. Myelin basal protein (MBP) and rabbit Immunoglobulin G (IgG) were included as negative (red) and positive (green) controls. Boxes of substituted amino acids causing considerable signal drops compared to the remaining peptides are coloured in orange.
(TIF)

S6 Fig Alanine scan of the identified epitope of NGO0170_1_3. The first and the last peptide are marked by a minus. The first peptide is additionally marked by a 1. The first fifteen spots correspond to the epitope sequence LLAREKIAAGATETI where each amino acid is substituted subsequently and each peptide is immobilised consecutively on one of the spots on the membrane. Spot 16 is the epitope peptide without substitution. Spot 17 a random peptide TESYLAQASAKMQQQ as negative control. Peptide spots 4 to 6 and 11 to 15 showed a dark staining comparable to the epitope peptide (spot 16) while peptides 1 to 3 and 7 to 10 showed a weaker staining. Spot 17 only showed a slight background stain.
(TIF)
