# Supplementary figures and images for: Epitope determination of immunogenic proteins of Neisseria gonorrhoeae
Source: PLoS One. 2017 Jul 19;12(7):e0180962. doi: 10.1371/journal.pone.0180962 (PMC5516995; doi:10.1371/journal.pone.0180962)

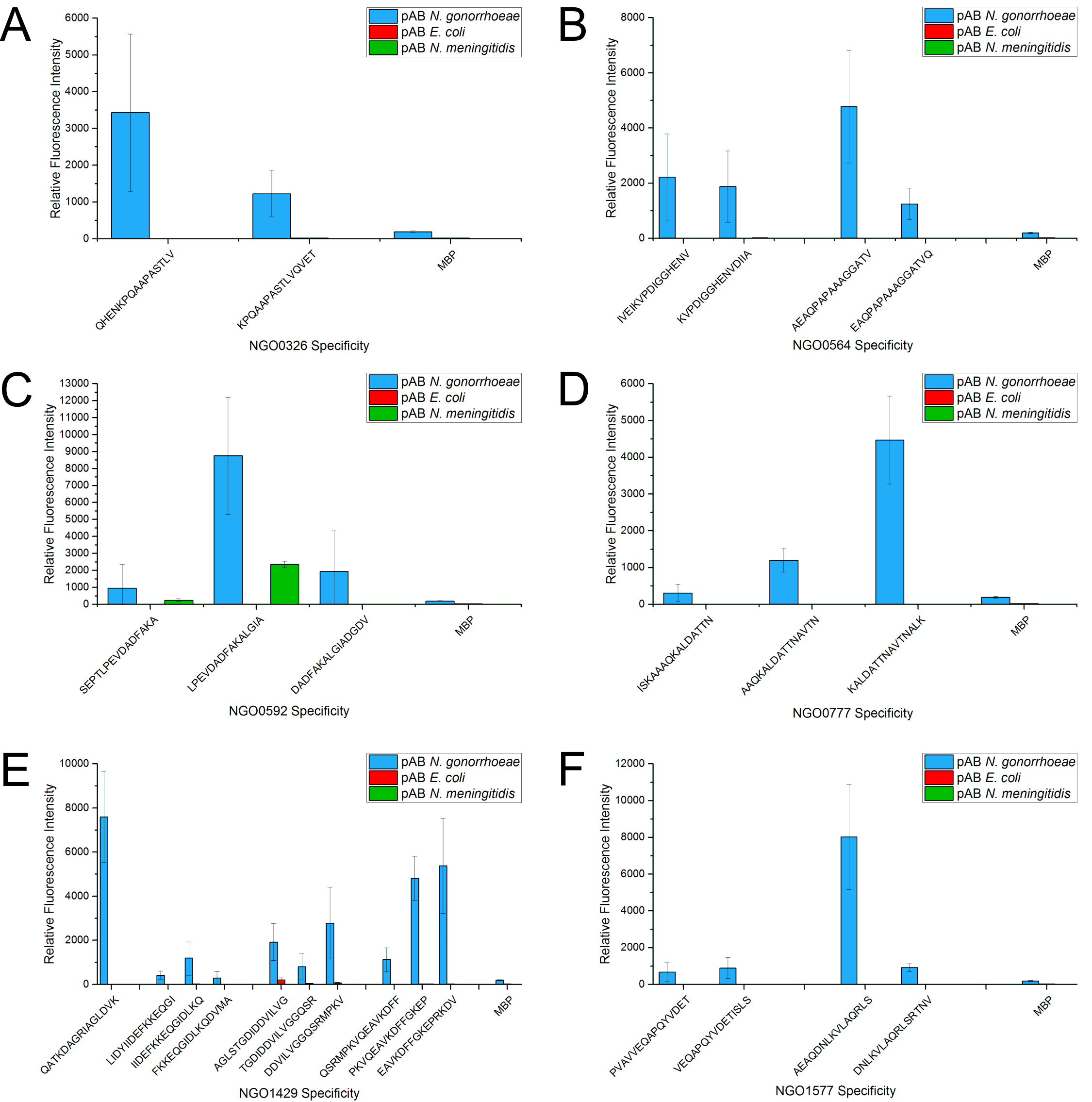

Supplement: S1 Fig — Specificity controls of the identified peptides with epitope features of A) NGO0326, B) NGO0564, C) NGO0592, D) NGO0777, E) NGO1429, F) NGO1577. Almost all peptides showed specific binding for anti-N. gonorrhoeae antibodies. Only two peptides of NGO0592 (C) showed unspecific binding to the polyclonal anti-N. meningitidis antibody correlating to approximately 25% of the signal obtained through specific binding. Minimal signal intensities could also be seen for two peptides of NGO1429 for the anti-E. coli antibody correlating to maximal 10% of the signal obtained through specific binding. (TIF) [file pone.0180962.s001.tif]

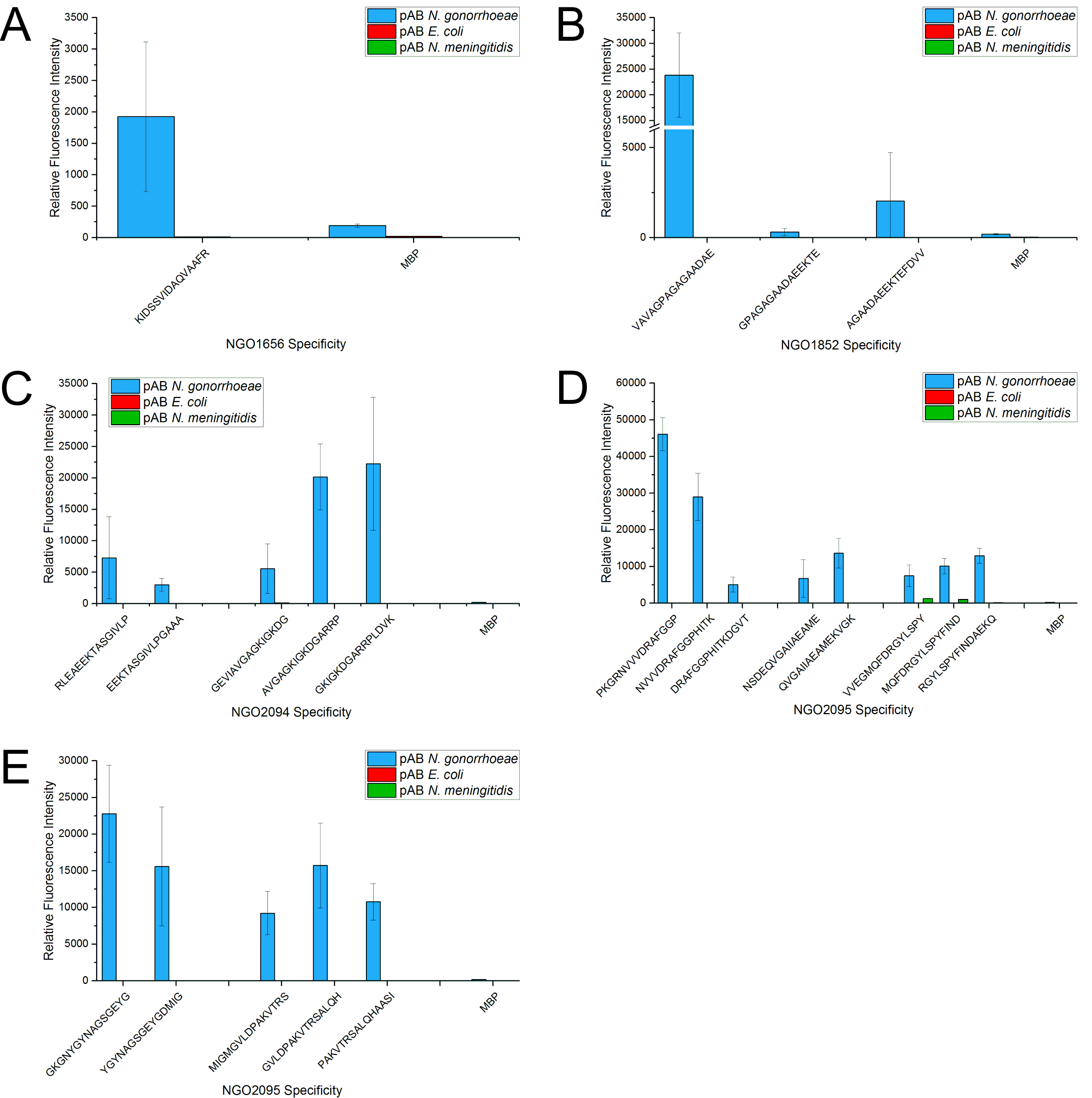

Supplement: S2 Fig — A) NGO1656, B) NGO1852, C) NGO2094, D) the first three potential epitopes NGO2095, E) further two potential epitopes NGO2095. Almost all peptides showed specific binding for anti-N. gonorrhoeae antibodies. Only two peptides of NGO2095 showed signal intensities for the anti-N. meningitidis antibody correlating to approximately 10% of the signal obtained through specific binding. (TIF) [file pone.0180962.s002.tif]

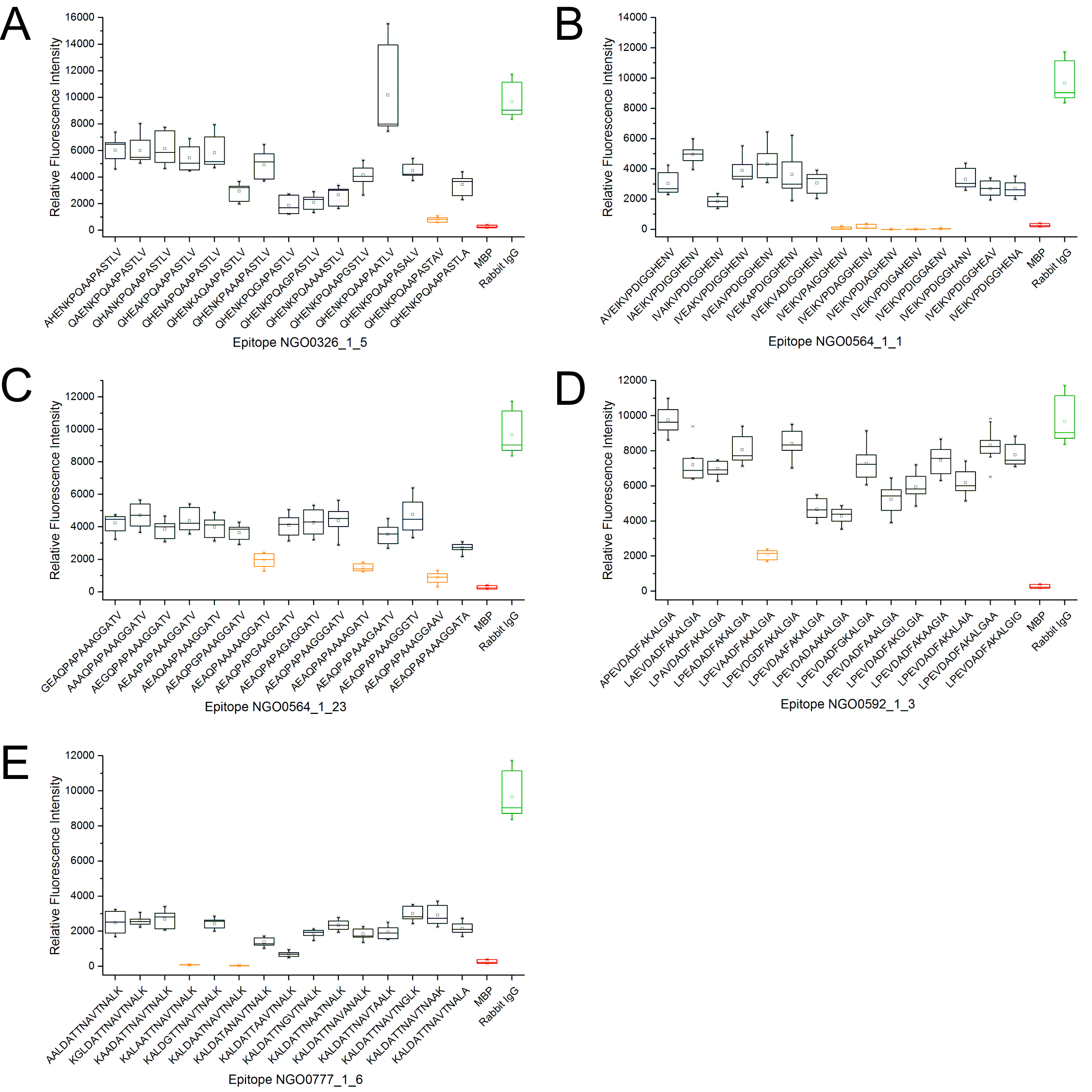

Supplement: S3 Fig — Alanine scans of the identified epitopes of A) NGO0326_1_5, B) NGO0564_1_1, C) NGO0564_1_23, D) NGO0592_1_3, E) NGO0777_1_6. The boxes embody 50% of the values, while the whiskers comprise 98% of the data. Outliers are marked with a small x. Median values are indicated as a horizontal line, while the mean values are marked by a small square. Myelin basal protein (MBP) and rabbit Immunoglobulin G (IgG) were included as negative (red) and positive (green) controls. Boxes of substituted amino acids causing considerable signal drops compared to the remaining peptides are coloured in orange. (TIF) [file pone.0180962.s003.tif]

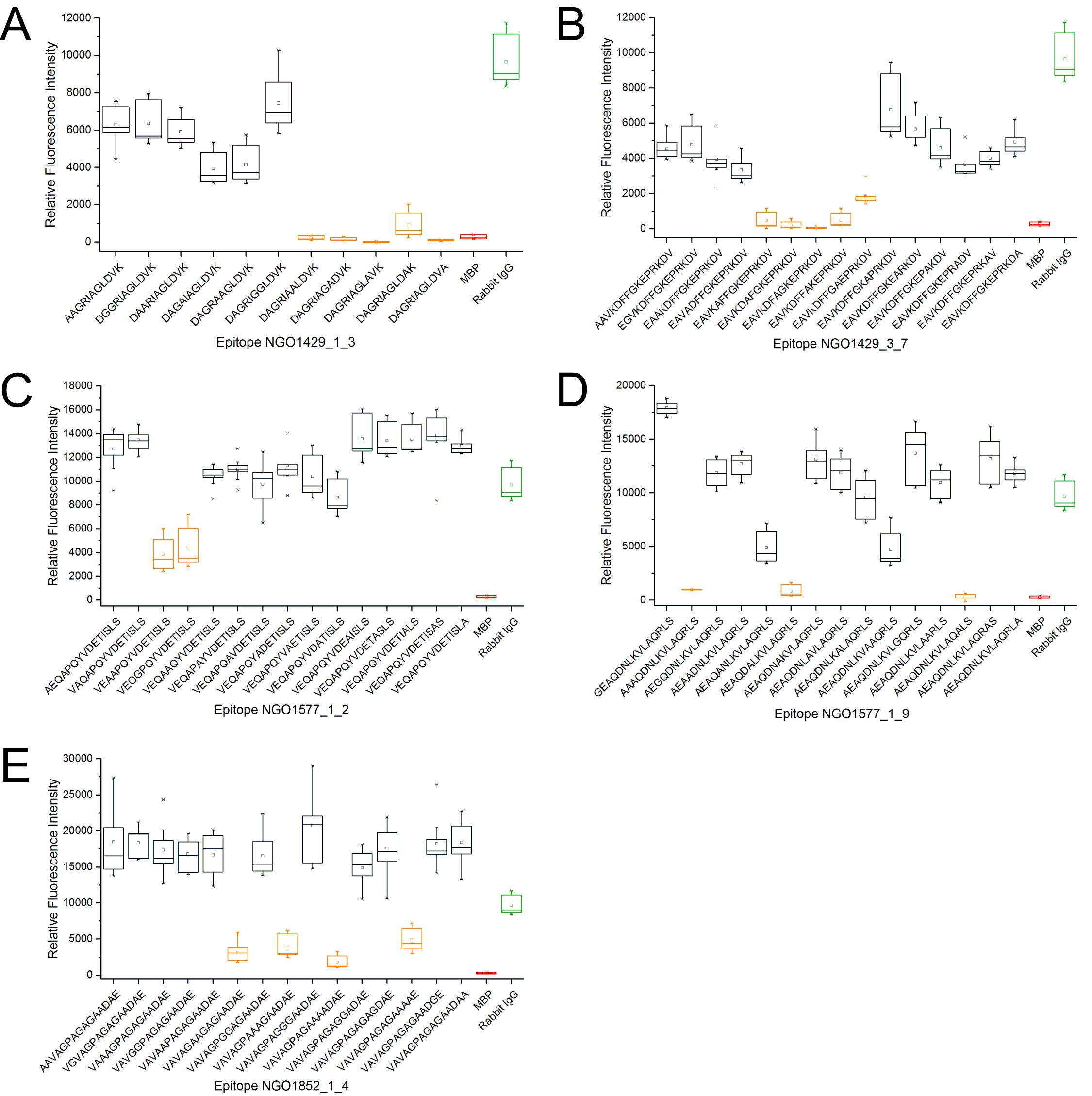

Supplement: S4 Fig — Alanine scans of the identified epitopes of A) NGO1429_1_3, B) NGO1429_3_7, C) NGO1577_1_2, D) NGO1577_1_9, E) NGO1852_1_4. The boxes embody 50% of the values, while the whiskers comprise 98% of the data. Outliers are marked with a small x. Median values are indicated as a horizontal line, while the mean values are marked by a small square. Myelin basal protein (MBP) and rabbit Immunoglobulin G (IgG) were included as negative (red) and positive (green) controls. Boxes of substituted amino acids causing considerable signal drops compared to the remaining peptides are coloured in orange. (TIF) [file pone.0180962.s004.tif]

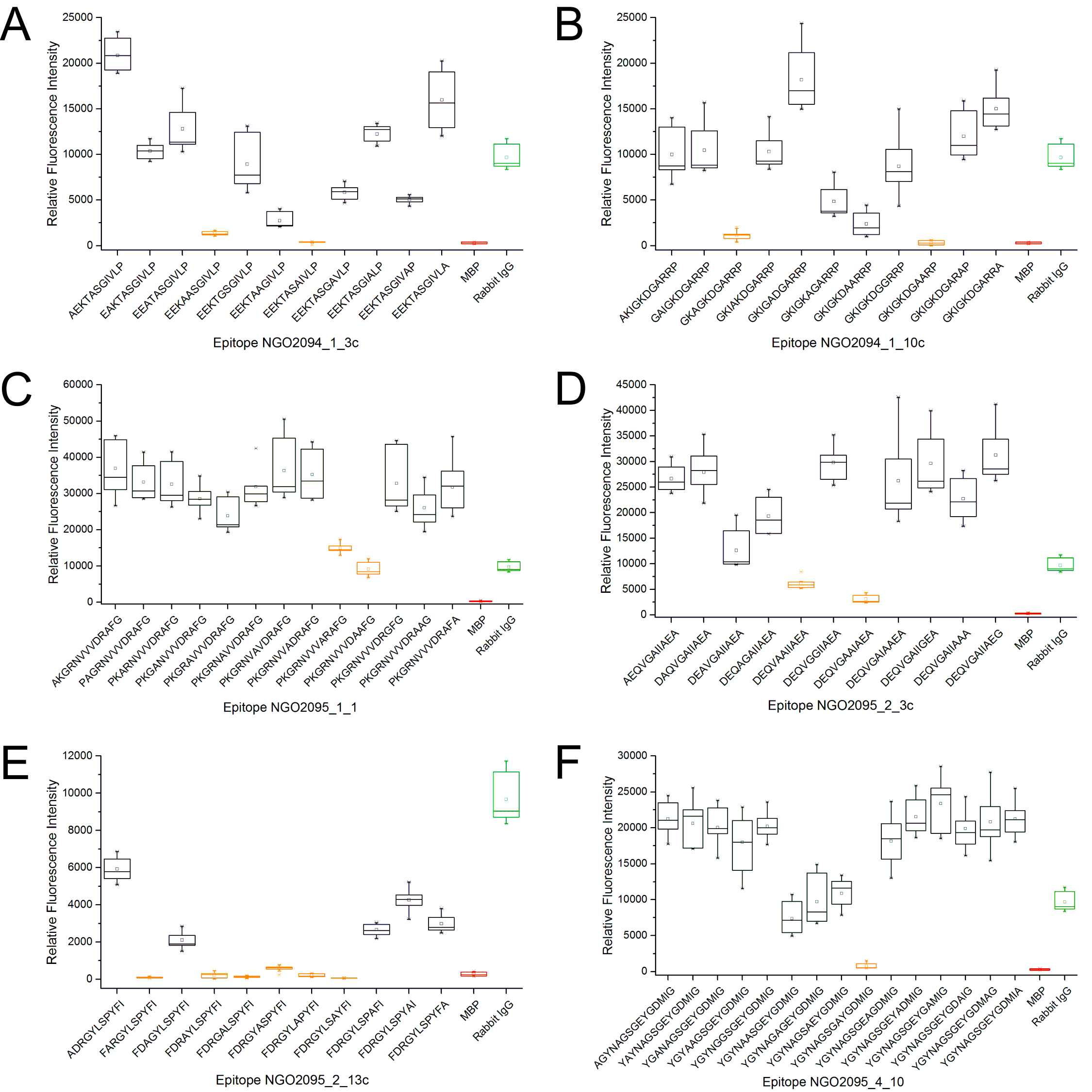

Supplement: S5 Fig — Alanine scans of the identified epitopes of A) NGO2094_1_3c, B) NGO2094_1_10c, C) NGO2095_1_1, D) NGO2095_2_3c, E) NGO2095_2_13c, F) NGO2095_4_10. The boxes embody 50% of the values, while the whiskers comprise 98% of the data. Outliers are marked with a small x. Median values are indicated as a horizontal line, while the mean values are marked by a small square. Myelin basal protein (MBP) and rabbit Immunoglobulin G (IgG) were included as negative (red) and positive (green) controls. Boxes of substituted amino acids causing considerable signal drops compared to the remaining peptides are coloured in orange. (TIF) [file pone.0180962.s005.tif]

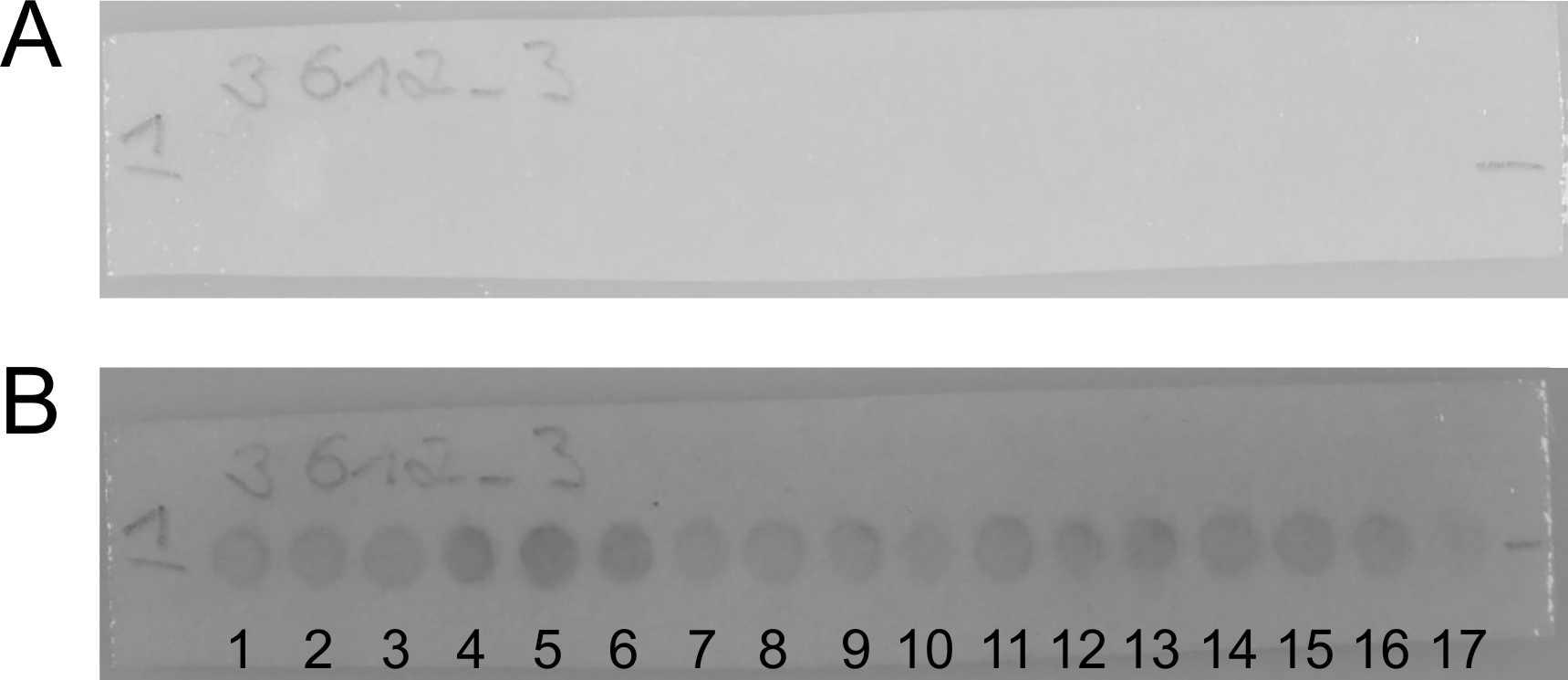

Supplement: S6 Fig — The first and the last peptide are marked by a minus. The first peptide is additionally marked by a 1. The first fifteen spots correspond to the epitope sequence LLAREKIAAGATETI where each amino acid is substituted subsequently and each peptide is immobilised consecutively on one of the spots on the membrane. Spot 16 is the epitope peptide without substitution. Spot 17 a random peptide TESYLAQASAKMQQQ as negative control. Peptide spots 4 to 6 and 11 to 15 showed a dark staining comparable to the epitope peptide (spot 16) while peptides 1 to 3 and 7 to 10 showed a weaker staining. Spot 17 only showed a slight background stain. (TIF) [file pone.0180962.s006.tif]
